# Multi-platform assessment of transcriptional profiling technologies utilizing a precise probe mapping methodology

**DOI:** 10.1186/s12864-015-1913-6

**Published:** 2015-09-18

**Authors:** Jinsheng Yu, Paul F. Cliften, Twyla I. Juehne, Toni M. Sinnwell, Chris S. Sawyer, Mala Sharma, Andrew Lutz, Eric Tycksen, Mark R. Johnson, Matthew R. Minton, Elliott T. Klotz, Andrew E. Schriefer, Wei Yang, Michael E. Heinz, Seth D. Crosby, Richard D. Head

**Affiliations:** Genome Technology Access Center, Department of Genetics, Washington University in Saint Louis School of Medicine, 660 S. Euclid Ave. Campus Box 8232, Saint Louis, MO 63110 USA

**Keywords:** Transcript pattern, Fold-change, RNA-seq, Microarray, TaqMan assay

## Abstract

**Background:**

The arrival of RNA-seq as a high-throughput method competitive to the established microarray technologies has necessarily driven a need for comparative evaluation. To date, cross-platform comparisons of these technologies have been relatively few in number of platforms analyzed and were typically gene name annotation oriented. Here, we present a more extensive and yet precise assessment to elucidate differences and similarities in performance of numerous aspects including dynamic range, fidelity of raw signal and fold-change with sample titration, and concordance with qRT-PCR (TaqMan). To ensure that these results were not confounded by incompatible comparisons, we introduce the concept of probe mapping directed “transcript pattern”. A transcript pattern identifies probe(set)s across platforms that target a common set of transcripts for a specific gene. Thus, three levels of data were examined: entire data sets, data derived from a subset of 15,442 RefSeq genes common across platforms, and data derived from the transcript pattern defined subset of 7,034 RefSeq genes.

**Results:**

In general, there were substantial core similarities between all 6 platforms evaluated; but, to varying degrees, the two RNA-seq protocols outperformed three of the four microarray platforms in most categories. Notably, a fourth microarray platform, Agilent with a modified protocol, was comparable, or marginally superior, to the RNA-seq protocols within these same assessments, especially in regards to fold-change evaluation. Furthermore, these 3 platforms (Agilent and two RNA-seq methods) demonstrated over 80 % fold-change concordance with the gold standard qRT-PCR (TaqMan).

**Conclusions:**

This study suggests that microarrays can perform on nearly equal footing with RNA-seq, in certain key features, specifically when the dynamic range is comparable. Furthermore, the concept of a transcript pattern has been introduced that may minimize potential confounding factors of multi-platform comparison and may be useful for similar evaluations.

**Electronic supplementary material:**

The online version of this article (doi:10.1186/s12864-015-1913-6) contains supplementary material, which is available to authorized users.

## Background

Gene expression microarrays have provided an efficient and cost effective means for estimating RNA levels and differences on a transcriptome scale for nearly two decades. Since their arrival, millions of biological samples have been processed and analyzed utilizing this workhorse technology providing a window to the inner workings of cells, tissues, and organisms. For many years microarrays remained the primary source of knowledge pertaining to large scale gene expression. However, within the last few years RNA-seq has arisen as a competitive technology [1, 2] that many believe has already displaced microarrays as the definitive source of high-throughput gene expression data.

While microarrays have performed admirably through their tenure, they do suffer from some inherit limitations [3, 4]. Primarily, a microarray is a closed system that requires prior knowledge of the RNA species to be measured. In short, you can only measure what you spot for, and you can only spot for what you know. Conversely, RNA-seq is an open system that can, in theory, measure any RNA species present within the system without prior knowledge of the transcriptome content. Furthermore, given that the sensitivity and dynamic range of RNA-seq are only limited by the depth to which an investigator is willing to sequence, it stands to reason that this RNA-seq technology would be superior in multiple ways. Based on the stated advantages it would seem there would be little reason for investigators to continue with the use of microarrays. In practice, however, the comparison of differences between the technologies turns out to be a bit more complex.

A number of publications have produced fundamental comparisons between RNA-seq and microarrays in an effort to illuminate the differences and similarities [5–11]. While these studies demonstrated substantial correlations between RNA-seq and microarray, they highlighted a number of advantages of RNA-seq over microarrays in the detection of differentially expressed genes. This seems to be particularly true for genes with low expression levels. However, many of these investigations were limited in the number of evaluated microarray platforms and/or the degree of bioinformatic comparison, likely due to the somewhat complicated nature of the bioinformatic appraisal. In addition, we have noted several large scale comparison studies, such as the MAQC-III multi-community efforts [12], the ABRF next-generation sequencing study [13], and Wang et al. study [14], but these have been primarily focused on the accuracy and reproducibility of multiple RNA-seq systems, protocols, and data processing pipelines. As a result, we sought to expand on the foundations laid by the prior work above, and a study was completed with the following elements. First, we compared four microarray platforms (1 Agilent (symbolized as AGLN), 2 Affymetrix (Gene1.0 and HTA2.0), and 1 Illumina (ILMN)) and two RNA-seq library preparation protocols (poly-A selection based Clontech (ClonTech) and rRNA depletion based Ribo-Zero (RiboZero) approaches). Second, we sought to perform the analysis of expression data in an apples-to-apples fashion by ensuring that entities being compared represented common transcript sets, or “transcript patterns”, for a given gene. As defined in the Method section, the transcript pattern was referred as to a subset of probe(set)s of specific genes that targeted a common set of transcripts across all microarray platforms. With these elements in hand, our primary goal was to determine if any particular performance advantages/deficiencies exist within a given platform or technology as a whole. Our secondary goal was to evaluate the utility of the transcript pattern approach for cross-platform and cross-technology comparisons. To reach these goals, we analyzed the data at multiple levels, particularly a refined level of transcript pattern data, and presented results in numerous aspects, including dynamic range, signal to background ratio, fidelity of raw signal and fold-change with sample titration at and across platforms, and validation with fold-change concordance of RNA-seq/microarray to the gold standard qRT-PCR (TaqMan).

## Results

### Multiple levels of gene and transcript data sets

**Fig. 1:**
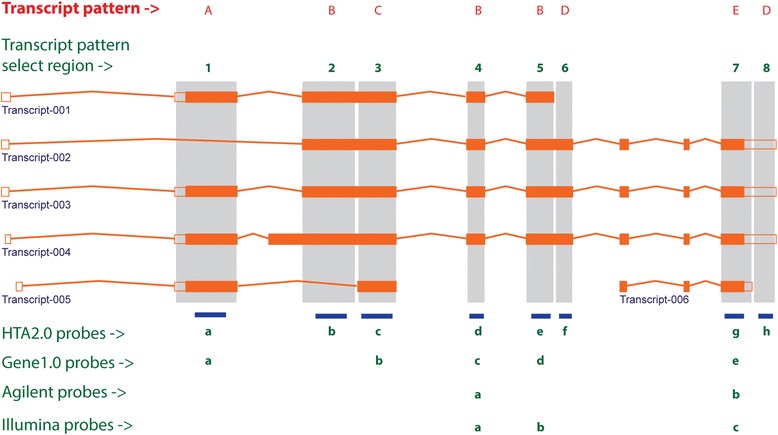
Diagram of transcript patterns defined in the current study. A transcript pattern select region covers a set of transcripts that share a certain exon or exon region recognized by a probe. The model gene in this diagram is TBP (exon/intron size modified for illustration purpose). If we consider, for example, Affymetrix probe a, which targets transcript pattern selection region #1 that covers a set of transcripts 001 and 003–005 but transcript 002 is excluded, defines the transcript pattern A. Thus, signals from Affymetrix HTA2.0 probes b, d, and e, from Gene1.0 probes c and d, from Agilent probes a, and from Illumina probes a and b will be used to summarize the expression level of the common transcript pattern B within a platform. Further, because the transcript patterns B and E are common across all microarray platforms they are kept in the transcript pattern derived subset data as two separate data points although they represent the expression level of the same specific gene (TBP)

Utilizing the approach described within the materials and methods, three different levels of gene/transcript data sets were defined and employed for the comparisons of differential expression across platforms (Table 1). The first level of analysis was performed on the complete data sets of each platform with no direct gene level comparison, and, as such, the number of data points measured varies. The second level of analysis was completed utilizing a subset of data comprising 15,442 RefSeq genes whose Entrez Gene ID were common across platforms. Finally, the most focused analysis was performed on a subset of data derived only from those microarray probe(set)s and RNA-seq exons that were mapped to identical transcript patterns for their given genes. A total of 7,043 transcript patterns representing 7,034 RefSeq genes were identified across the 6 platforms and protocols that met the defined criteria (Fig. 1). This subset of 7,034 RefSeq gene data was summarized from transcript pattern mapped probe(set)s and RNA-seq exons using the Tukey’s Bi-weight algorithm.

**Table 1:** Number of features analyzed in comparisons for all 6 platforms/protocols

Platforms /Protocols	# Features at probe(set) & exon level	# Non-control features at probe(set) & exon level	# Features at gene & transcript cluster level	# Genes in RefSeq & Ensembl gene database	# Detected genes & transcript clusters *	# Genes & transcript clusters for 15442 common RefSeq genes	# Probe(set)s & exons for transcript pattern restricted 7034 RefSeq genes	# Probe(set)s & exons for transcript pattern not restricted 7034 RefSeq genes
AGLN	43,376	41,000	29,066	24,961	22,056	15,442	8,668	11,453
Gene1.0	257,430	253,002	28,869	20,796	28,105	16,516	11,449	84,196
HTA2.0	914,585	573,909	67,526	25,195	67,309	16,400	14,908	138,484
ILMN	47,306	47,214	34,589	31,320	23,377	15,442	8,539	11,414
ClonTech	1,298,791	1,298,791	62,893	49,085	43,266	15,594	16,470	334,318
RiboZero	1,298,791	1,298,791	62,893	52,865	48,378	15,594	16,470	334,318

Notes:

1. AGLN: 41,000 probes represent 29,066 genes by GeneSymbol & SystematicName; 29,066 “genes” are composed of 24,961 entries with symbols and 4,105 with Agilent probe_ids only

2. Gene1.0: 253,002 probesets represent 28,869 genes by Affymetrix “transcript_cluster_id” (TC); 28,869 TCs are composed of 20,796 genes with symbols and 6,209 without symbols (NetAffx na33.2)

3. HTA2.0: 573,909 probesets represent 67,526 genes by Affymetrix “transcript_cluster_id” (TC); 67,526 TCs are composed of 25,195 genes with symbols and 40,696 without symbols (NetAffx na33)

4. ILMN: 47,214 probese represent 34,589 Illumina named “genes”, of which 31,320 have official gene symbols and 3,269 labeled with Unigene_ids (Hs.xxxxxx)

5. ClonTech: 1,298,791 exons represent 62,893 ensembl genes (ENSGs) in R72 database; 62,893 down to 49,085 ENSGs with at least 1 read in any of 5 samples

6. RiboZero: 1,298,791 exons represent 62,893 ensembl genes (ENSGs) in R72 database; 62,893 down to 52,865 ENSGs with at least 1 read in any of 5 samples

*A “detected” call for AGLN at gene level and for Gene1.0 and HTA2.0 at transcript cluster level was made if any probe(set) was “detected” in any sample by *p* < 0.05 (AGLN) or *p* < 0.01 (Gene1.0 & HTA2.0); and for RNA-seq data the detection calls were made if any samples had a cpm >0.25. ILMN data have “detected” calls by *p* < 0.05 at both probe and gene levels

### Assessment of signal and dynamic range

**Fig. 2:**
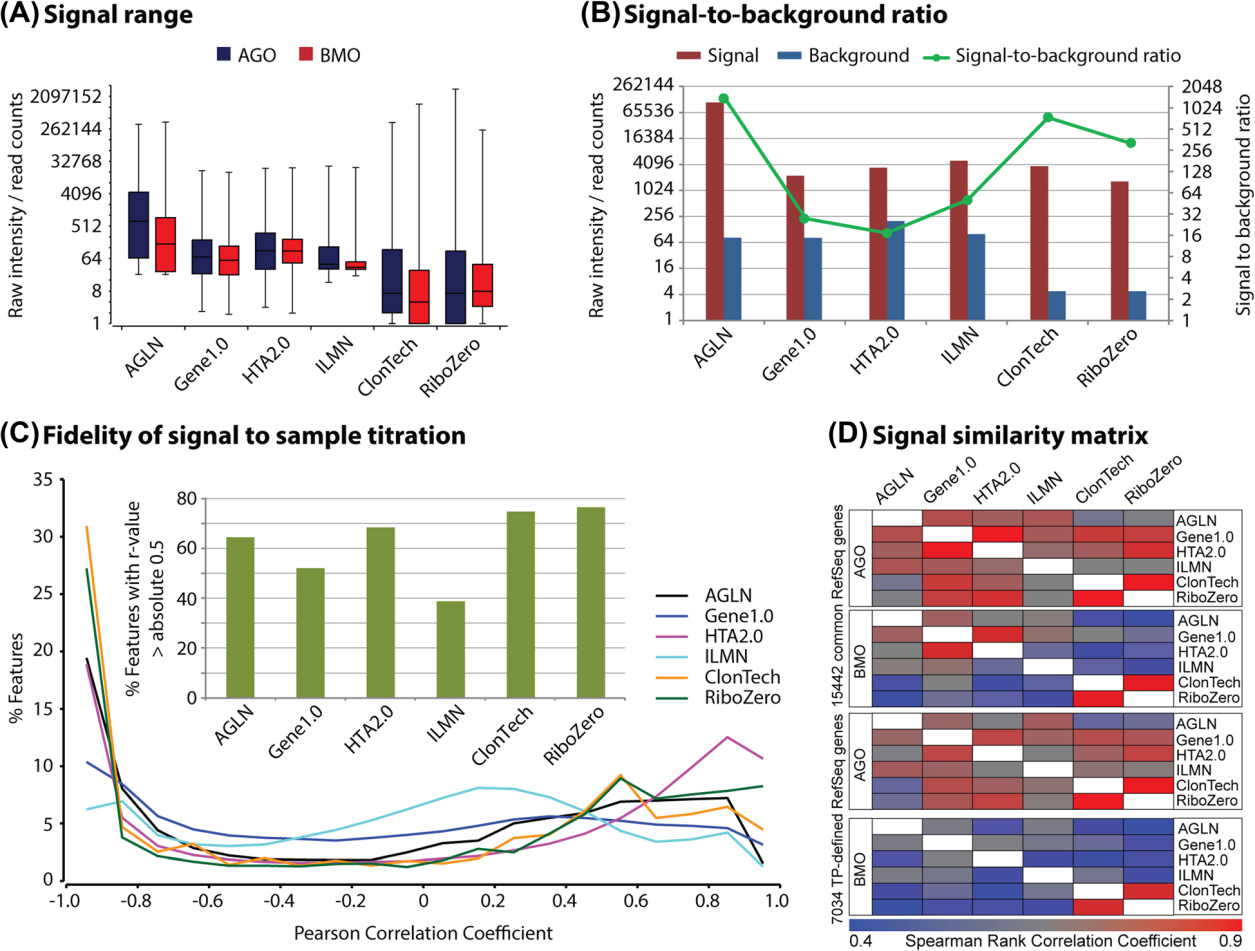
(**a**) raw signal range of two pure RNA samples (AGO and BMO) with the entire data set, represented by Box-Whisker plot (max, 75 %, median, 25 % and min); (**b**) signal-to-background ratio, indicated by 99 percentile of mean signal and background values determined with entire set of raw data in all 5 RNA samples; (**c**) fidelity of signal to sample titration by correlation, showing full distribution of coefficient of correlation between signal and titration across the 5 RNA samples in the entire set of raw data, with the emphasis on the percent of features (probes/probesets for microarray, genes for RNA-seq) with a correlation coefficient greater than absolute 0.5; (**d**) signal similarity matrix of AGO and BMO samples across all 6 platforms were generated with Spearman rank correlation using signal/count data of RefSeq gene symbol aligned 15442 genes and transcript pattern defined 7034 genes

The expression data generated on all platforms were using the same 5 RNA samples (an Agilent Universal Human Reference RNA (labeled as AGO), a normal pooled bone marrow RNA (labeled as BMO), and 3 of AGO/BMO mixed samples at a ratio of 1:4, 1:16, and 1:64 (labeled as AG1BM4, AG1BM16, and AG1BM64) (Table 2). These 5 RNA samples formed a titration in terms of the amount of pure AGO or BMO in each sample. The percentage of bone marrow RNA contained in the samples AGO, AG1BM4, AG1BM16, AG1BM64, and BMO was 0 %, 75 %, 93.75 %, 98.4375 %, and 100 %, respectively. The two original non-mixed RNA samples (AGO and BMO) are expected to have the greatest diversity in gene expression levels. As such, these samples were selected to calculate minimum, maximum, median, 25 percentile, and 75 percentile of non-normalized raw signal values for a visual inspection of the overall signal distribution across platforms. These data are intended to provide a raw estimation as to the dynamic range differences of the platforms, and as such, are not normalized. The resulting data describe the basic signal range of each platform (Fig. 2a). Additionally, we computed the average 99^th^ percentile signal value of all 5 RNA samples along with the mean background value to calculate the signal-to-background ratio for all platforms (Fig. 2b). For RNA-seq data, non-normalized raw count data were used in the calculation of signal range. Technically, RNA-seq data have no background. However, it was noted that a number of genes demonstrated a non-zero count in the 3 mixed RNA samples (AG1BM4, AG1BM16, and AG1BM64) but had no counts at all in their parent RNA samples (AGO and BMO), indicating some degree of noise in the RNA-seq data. We have noted that several studies have proposed the methods for calculation of a quasi-background for RNA-seq data [15, 16], but they utilized either reads in intergenic regions or technical replicates, which are not available in our data. Thus, we sought to utilize those particular “noise” genes for calculation of a quasi-background value for our RNA-seq data. These RNA-seq genes accounted for less than 5 % of all genes and had a sequence read count ranged from 1 to 12 that represents a common observation of the lower end read count variation, particularly with common read depths such as those used here. With this subset of data, the quasi-background value for each RNA-seq protocol as a whole was represented by the median of 99th percentiles of raw counts in the 3 RNA samples. While it could be argued this is a somewhat artificial construct for background in RNA-seq data, the resulting signal-to-background ratios in the present study were quite indicative of platform performance: the larger the signal-to-background ratio, the better the concordance with the measures of titration response and TaqMan validation (see following result sections). Amongst the microarray platforms, Agilent demonstrated the largest signal range and signal-to-background ratio and Illumina had the least values of these metrics, as seen in Fig. 2a-b. Within the RNA-seq protocols, ClonTech appeared to produce signal ranges and “quasi-signal-to-background” ratios that were better than RiboZero.

**Table 2:** Samples used in the study

Sample Name	Ratio of AGO:BMO	% BMO	Replicate						
			AGLN	Gene1.0	HTA2.0	ILMN	ClonTech	RiboZero	TaqMan
AGO	1:0	0	1	2	2	2	1	1	6
AG1BM4	1:4	75	2	2	2	3	1	1	6
AG1BM16	1:16	93.75	2	2	2	2	1	1	6
AG1BM64	1:64	98.4375	2	2	2	2	1	1	6
BMO	0:1	100	1	2	2	3	1	1	6

### Fidelity of signal with sample titration and signal similarity across platforms

A linear regression analysis was performed between the sample titration and the signal or read count for each feature (probe, probeset, exon, or gene) within each platform. The overall distribution of the coefficient of correlation can be seen in Fig. 2c where Gene1.0 and ILMN data showed the least amount of features with a coefficient of correlation greater than absolute 0.8 and more features with the coefficient of correlation between −0.4 to +0.4. To set a single comparison point, a correlation coefficient of absolute 0.5 was defined to indicate a qualitative fit to the titration series above and the percent of features exceeding this level was calculated. Based on this assessment the relative fidelity level of each platform can be seen in the bar chart of Fig. 2c. Furthermore, a Spearman rank correlation analysis was conducted across all 6 platforms using gene symbol aligned 15442 RefSeq genes as well as 7034 transcript pattern defined genes. As shown in Fig. 2d, a higher overall similarity was seen in AGO than that in BMO, and so was in the 15442 set than that in the 7034 set. Platform-wise, data between two RNA-seq protocols and between two Affymetrix platforms were quite similar. As expected, data between RNA-seq and microarrays were less similar than microarray-to-microarray, likely due to their distinct data distribution (Poisson vs. Gaussian).

### Fidelity of fold-change with sample titration at and across platforms

**Fig. 3:**
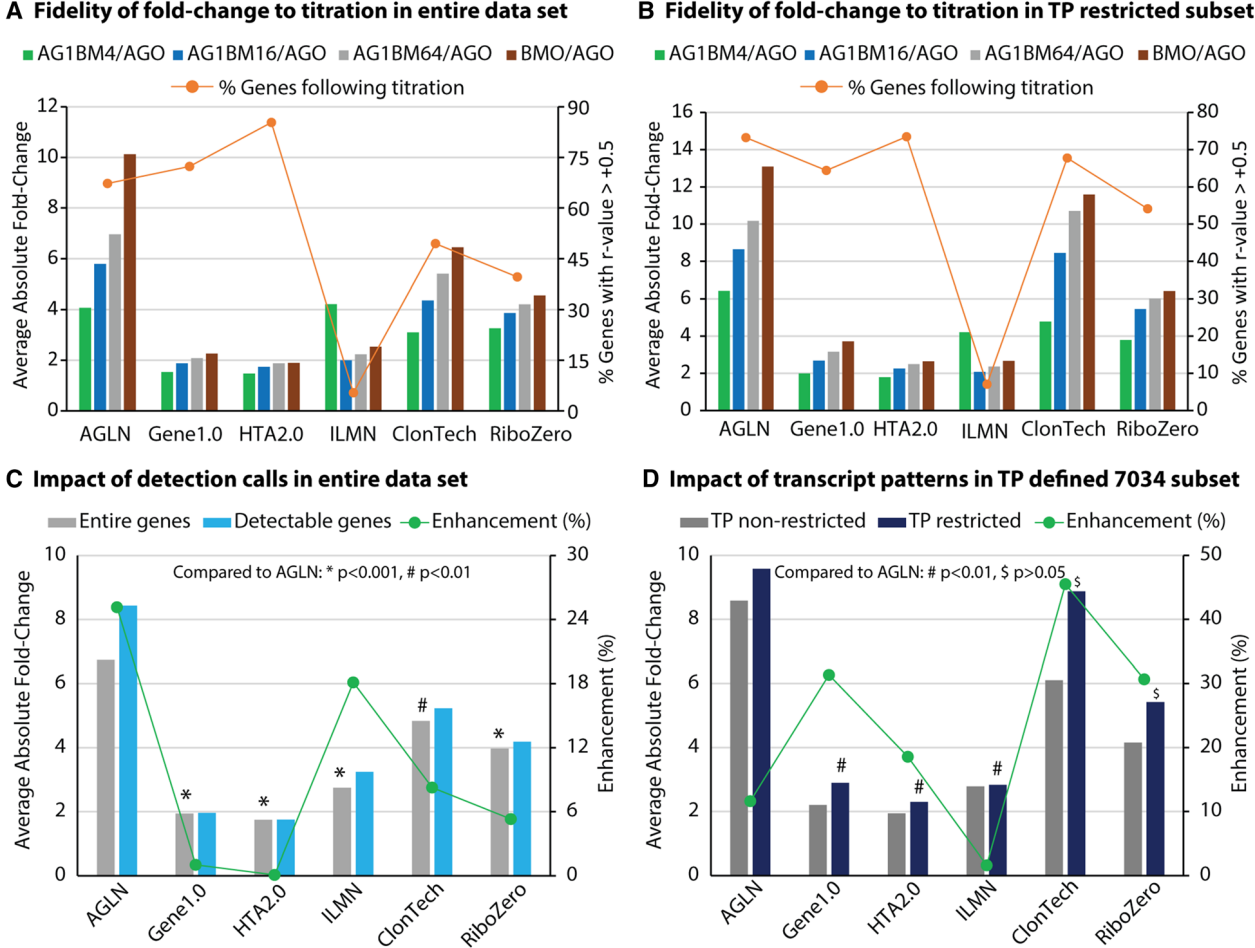
Bar charts for platform comparisons on magnitude of differential expressions determined by average absolute fold-change. Average absolute fold-change was analyzed for each titration across all 6 platforms in entire data set (**a**) as well as in transcript pattern (TP) restricted 7,034 subset (**b**). To ascertain the magnitude of differential expression for a platform as a whole, the 4 average absolute fold-changes of the full titrations were averaged in both entire genes and detectable genes in the entire data set (**c**), as well as in TP non-restricted and restricted 7,034 RefSeq genes subsets (**d**). To gauge platform fidelity level in fold-change along sample titrations, percent of genes with a Pearson correlation > +0.5 was indicated in the panels (**a**) and (**b**). In addition, the fold-change enhancement was indicated with dotted lines in green in panels (**c**) and (**d**) that was determined as the difference in average absolute fold-change between the bar elements from left to right for each platform. Moreover, the statistics were placed in the panels (**c**) and (**d**) for the difference in average fold-change from AGLN to the other platforms for the entire set of data and for the TP-defined subset of data. When compared to AGLN, the average absolute fold-change was significantly lower in all platforms (*p* < 0.01–0.001) in the data set for entire genes, and such difference was statistically significant to 3 microarray platforms (*p* < 0.01) but not to the RNA-seq protocols (*p* > 0.05) in the TP restricted 7,034 subset

An assessment of overall fold-change magnitude was completed at sample titrations and at platforms as a whole, utilizing both the entire data sets and the transcript pattern restricted subsets. The average absolute fold-change was calculated at each of the four sample titrations with all of the above data sets for each platform (Fig. 3a-b), and these values at each of the sample titrations were further averaged to represent the overall fold-change of a platform (Fig. 3c-d). The variability of fold-change data was assessed as well and is provided in Additional file 1: Figure S1. Technical replicates were employed for most of the microarray data; however, as average fold-change and overall fold-change data were utilized for cross-platform comparison, individual sample variation within the RNA-Seq data should have minimal impact on the overall results. In order to gauge the platform difference in the fidelity of fold-change with sample titration, we made a Pearson correlation test on the absolute fold-change values along the 4 titrations. The percent of genes that had r value greater than positive 0.5 was calculated for each platform in each of the 2 data sets (Fig. 3a-b). In Fig. 3a-b, a clear ascending trend across the 4 sample titrations towards the pure sample comparison of BMO vs. AGO can be seen for the majority of the platforms, and the percent of genes following the titrations was comparable across most of platforms (40-85 % in the entire sets and 54-73 % in the 7,034 subsets). A number of observations are worthy of note here. First, the AGLN microarray and ClonTech RNA-seq protocol appear to produce differentials of substantially greater magnitude than the other platforms in both the entire set and subset comparisons. Second, while both Affymetrix platforms trend appropriately, there is a clear degree of compression by comparison, i.e. a smaller fold-change that was observed in Affymetrix platforms than those in Agilent and RNA-seq methods. To improve fold-change issues in HTA2.0 signal data, Affymetrix recently released a new algorithm called “Signal Space Transformation” (SST) [17]. The SST did increase the overall absolute fold-changes of HTA2.0 arrays to a level that was quite comparable to Agilent and RNA-seq methods (Additional file 2: Figure S2). Third, the Illumina microarray platform, strikingly, did not trend well with the sample titration as the absolute fold-change values that followed the sample titrations were observed in only 5-7 % of genes. This observation was lower than that for Agilent arrays (67-73 %). Finally, when comparing the 7,034 gene transcript pattern restricted sets, a high degree of similarity in average absolute fold-change was seen apparently between AGLN and ClonTech (Fig. 3b). To remove any potential bias of undetected genes/transcripts on the fold-change assessment, the comparison for the entire data sets was done with undetected genes removed from the analysis (Fig. 3c). While the average absolute fold-change increased to certain degree for most of platforms, the relative differences between platforms were highly conserved. Similarly, the fold-change assessment for the transcript pattern restricted subset was repeated utilizing all platform-designated probe(set)s for the 7,034 genes to determine if the similarity is simply an artifact of the gene subset measured. In this case, it can be seen that the close alignment of overall fold-change between AGLN and ClonTech was the most highly preserved (Fig. 3d). In order to quantify the impact of both detection call and transcript pattern, the enhancement in fold-change magnitude was calculated. For the entire data set, the calculation was completed using data from all genes and from only detectable genes. For the transcript pattern defined subset, the calculation was done using data from all probe(set)s on arrays and all exons in RNA-seq for the 7,034 genes and from only those probe(set)s and exons mapped to identical transcript patterns for the same 7,034 genes (i.e. transcript pattern restricted). As such, the fold-change enhancement by the detection call ranged from 0 –25 % (10 % on average); and it was 2 –45 % (23 % on average) by the transcript pattern (Fig. 3c-d). These results implicated the usefulness of detection call and particularly the transcript pattern approach in eliminating confounding factors when making cross-platform comparisons.

### Assessment of cross-platform fold-change correlation

**Fig. 4:**
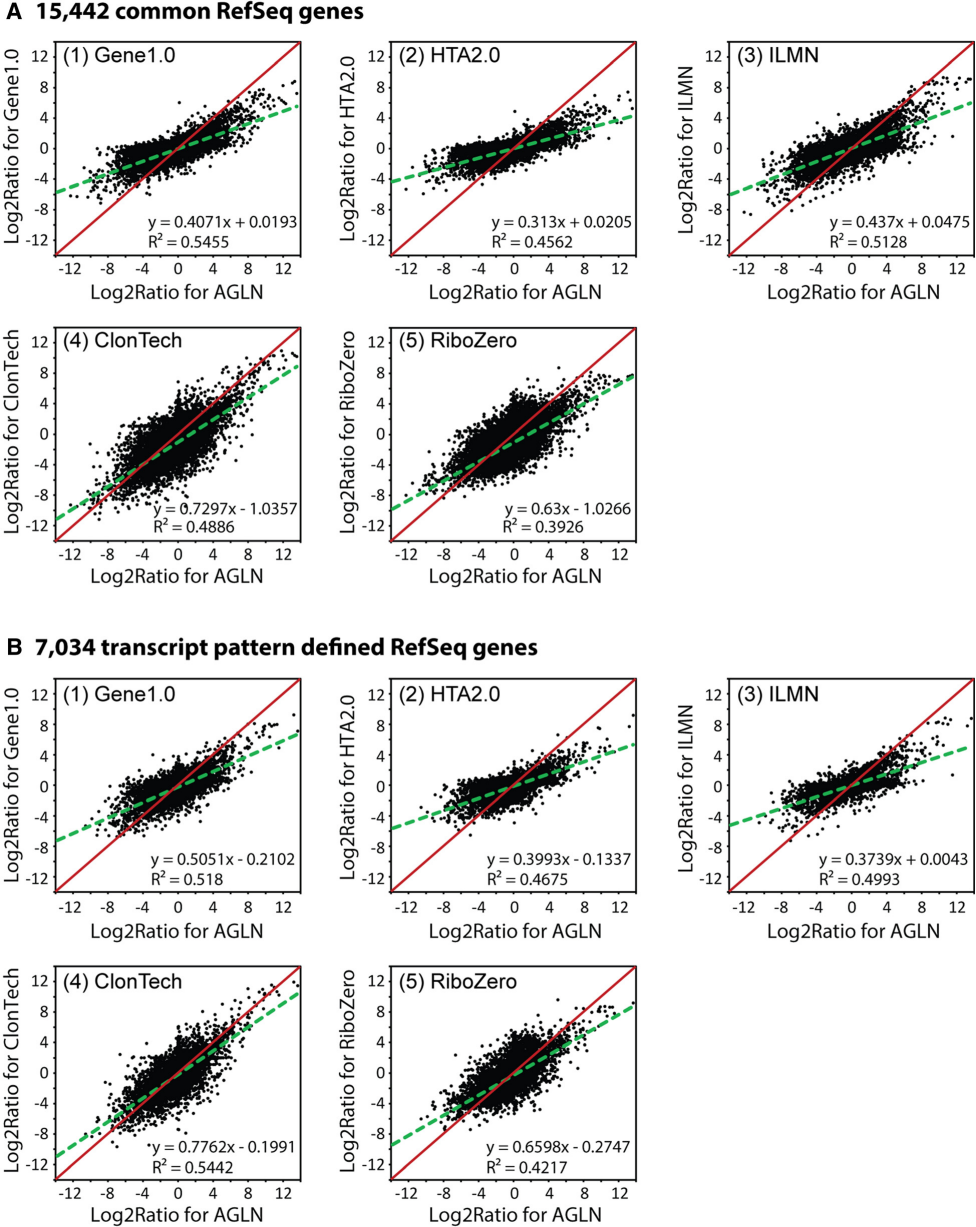
Scatter plots for log2 ratio of the BMO vs. AGO contrast for platform comparisons using data of (**a**) 15,442 common RefSeq genes and (**b**) transcript pattern restricted 7,034 RefSeq genes, each against AGLN. The dotted green lines are trend lines by linear regression, and the red lines are diagonal lines of the frames. The deviation of green lines from red lines indicates the degree of fold-change compression that can be quantified by slope values in the equations

A complete log2 AGO/BMO ratio set was generated for each platform/protocol. This particular comparison was employed as it produced the greatest overall range of differentials. As the AGLN platform appeared to demonstrate both the largest absolute fold-changes and maintained strong fidelity with the sample titration, it was utilized as the standard to investigate the degree of cross-platform correlation. Scatter plots were generated for all platforms/protocols comparisons in the log2 ratio space (Fig. 4 for comparisons to AGLN, and Additional file 3: Figure S3 for all other comparisons). Furthermore, based on the linear fit, the slope and correlation coefficients (R^2^) were also calculated. While the R^2^ provides a basic estimate for the overall correlation, the slope presents a rough indication as to the degree of compression observed within each platform as compared to AGLN. Based on the measured slope (Fig. 4), we observed a 27 –69 % (50 % on average) fold-change compression in the entire dataset, and a 22 –63 % (46 % on average) fold-change compression in the transcript pattern defined subset data (calculated using formula: (1-slope)*100). Generally speaking, the AGLN and RNA-seq platforms clearly demonstrated a substantially greater range of log2 ratios regardless of the comparative gene set examined. Furthermore, a somewhat improved fit between AGLN and RNA-seq, ClonTech in particular, was observed with the transcript pattern restricted 7,034 gene subset, similar to the observation in Fig. 3a-b.

### TaqMan qRT-PCR assessment of fold-change accuracy

**Fig. 5:**
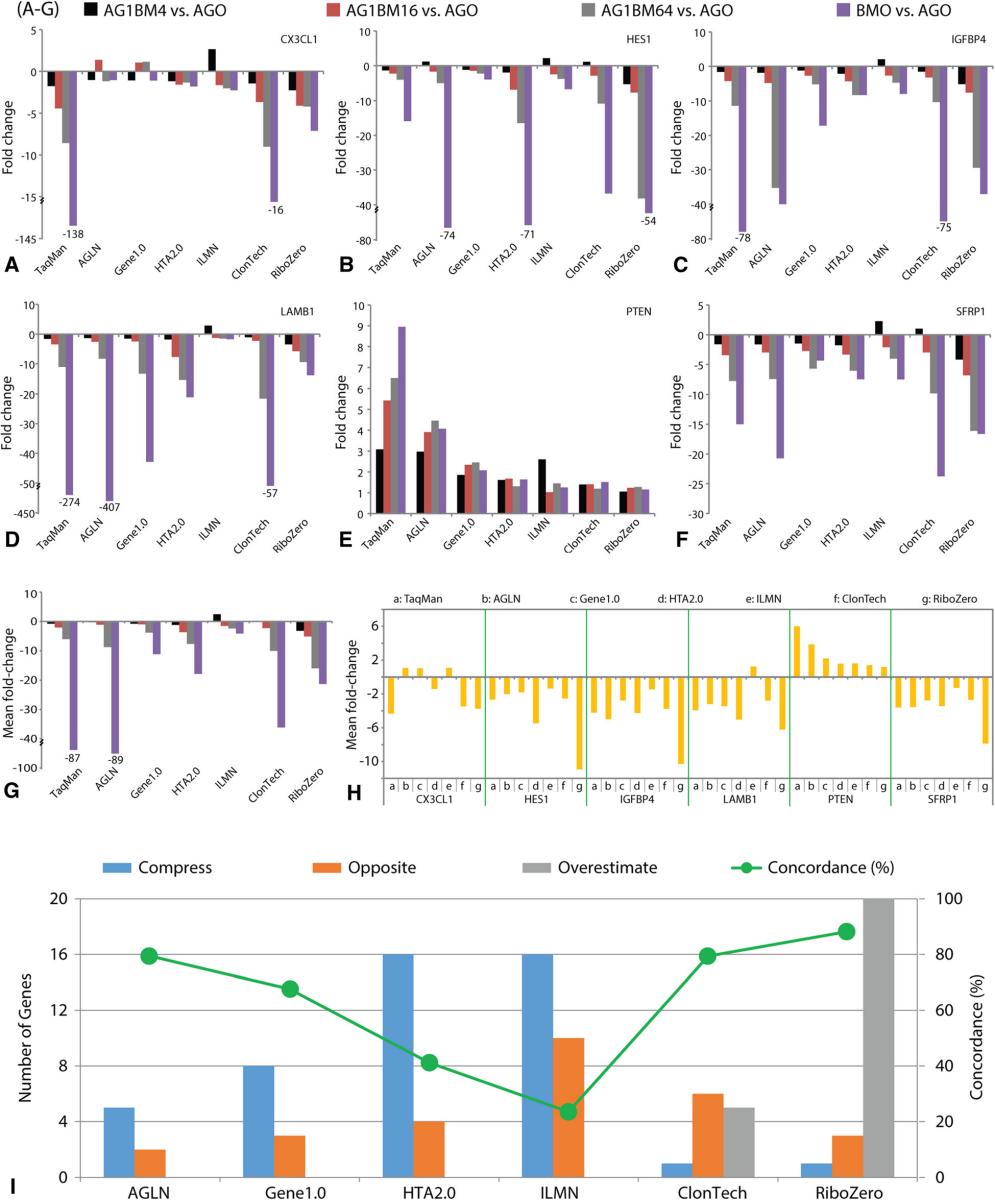
TaqMan qRT-PCR validation. (**a**-**f**) fold-change data derived from titrated samples. 6 genes were selected because the TaqMan probes targeted the same transcript pattern as did the microarray probe(set)s; (**g**) mean fold-change of the 6 genes across platforms; extreme values in (**a**-**d**) and (**g**) were indicated with broken y-axis and actual data; (**h**) mean fold-change of the 4 titrations across platforms; (I) Concordance of fold-change between TaqMan qPCR (X) and microarrays/RNA-seq protocols (Y), 4 different calls were made: compress, opposite, overestimate, and concordant. When two compared fold-changes are in the same direction but the ratio of X/Y greater than or equal to 2, a value of “compressed” is assigned. Similarly, if the fold-change ratio of X/Y is less than or equal to 0.5 the comparison is deemed “overestimate”. Fold-change ratios between these values are deemed “concordant”. When two fold-changes are not in the same direction and either of them is greater than 2 or less than 0.5, the comparison is determined to be “opposite”. Concordance rates were calculated by number of genes with “concordant” and “overestimate” calls divided by the total genes analyzed

The 48 genes were previously profiled with TaqMan assays in the pure BMO and AGO samples as part of an unrelated project. The BMO/AGO fold-change of these 48 genes ranged from 0–167 folds. The data for these 48 genes were utilized as a gold standard qRT-PCT comparator as they provided a diverse data set of both changing and unchanging genes. Of these 48 genes, 35 were in the 15,442 RefSeq gene set and had reliable Ct values for comparison in all 5 RNA samples in the current study. *GUSB* was utilized as a housekeeping gene for normalization and the PCR amplicon locations for the remaining 34 comparator genes were then investigated for overlap with regions that were restricted by the transcript patterns. As 6 of the 34 genes assayed had PCR probe targets that overlapped with the common transcript patterns from the other platforms, their data were analyzed across the full titration as seen in Fig. 5a-f. Mean fold-changes of the 6 genes and of the 4 titrations are summarized in Fig. 5g-h, respectively. While large fold-change variations across platforms, particularly in 3 of the 6 genes (*HES1*, *IGFBP4*, *LAMB1*), were detected when the pure bone marrow was compared to pure Agilent Human Universal Reference RNA (i.e. BMO vs. AGO), the AGLN platform appeared to produce the highest degree of fidelity overall across the titration series. The overall fold-change concordance, as defined in the methods section, among the 34 genes ranged from 24  to 88 % between TaqMan and microarray/RNA-seq assays (Fig. 5i). Agilent microarray as well as the two RNA-seq protocols demonstrated the highest degree of concordance (80 % or higher); whereas Illumina and HTA2.0 microarrays showed the lowest similarity (40 % or lower).

## Discussion

The current study has assessed the differences and similarities in performance between 2 RNA-seq and 4 microarray protocols/platforms and further compared these to TaqMan qRT-PCR assay. Before delving into a discussion of the comparative results, a number of items should be addressed as to how the platforms were tested. The sequencing for the RNA-seq protocols targeted a read depth of 25–30 million reads per sample. While this is not a particularly deep level of sequencing, we felt it was appropriate for the purpose of comparison, as reported in Zhao *et al.* study [10] that determined that a minimal 14 million of reads will be able to achieve the same gene detection levels as a standard microarray. Another note with regards to RNA-seq is that the ribosomal depletion method used was EpiCenter’s Ribo-Zero kit. This study was completed prior to the release of their RiboGold kit that has replaced Ribo-Zero within our lab. However, the level of ribosomal contamination in the samples from RiboZero protocol was low and, as such, likely had little impact on the final results. In contrast to the fairly standard approaches used to generate RNA-seq data, there were significant alterations to multiple microarray protocols. This is particularly true for the Agilent array platform that performed so well across each of the comparator categories. While the microarrays and scanner are off-the-shelf issue, the labeling protocol used for the Agilent arrays was custom, utilizing an adaptation of the Kreatech ULS^™^ labeling kit. This kit has demonstrated more efficient label incorporation than the standard Agilent protocol based on numerous prior comparisons completed in our lab, and as such, it was utilized as the protocol for this study. The improved efficiency, combined with the Agilent scanner’s greater dynamic range yields a full log unit increase in sensitivity as compared to other array platforms (Fig. 2). This enhancement in range and sensitivity yields results comparable to RNA-seq at the sequencing depth used for this study. Another deviation from standard protocol was the substitution with NuGEN Ovation PicoSL WTA V2 kit for Gene1.0 arrays which was originally adapted in the lab for cost considerations and was subsequently expanded to applications with partially degraded RNAs and/or low mass. In our hands the NuGEN kit is quite comparable to Affymetrix standard WT-plus RNA amplification reagents (a Life Technologies Ambion product).

A number of studies [5–10, 14] have explored the similarities and differences in raw signal intensity and the overlap of differentially expressed genes identified between RNA-seq and microarrays. Both of these components are quite relevant for any analytical comparison of the given technologies. However, given the substantial number of TaqMan assessments combined with the RNA titration data available, we also sought to investigate the performance of the platforms in accurately assessing fold-change estimates. Furthermore, the cross-platform comparisons in all prior studies have been centered on gene name annotation, i.e. matching entities in comparison by gene symbol, Entrez Gene ID, or other gene identifiers. In fact, there are numerous factors to consider when determining how to compare gene expression data across different technological platforms. First, not all genes or transcript species will be captured by all platforms. For example, ribosomal depletion methods for RNA-seq will measure SNO and microRNAs that would be lost using a poly-A enrichment approach. Thus, a simple one-to-one mapping of all transcripts across all platforms is not possible. Second, the genomic location of microarray probes from different platforms may measure signal from different sets of transcripts for a given gene. Therefore, matched signal comparison by common RefSeq annotation at the gene level may not be entirely accurate. However, it is possible to directly map microarray probe(set)s based on genome coordinates to refine a subset of probe(set)s that target common sets of transcripts for specific genes. Therefore, we introduced the concept of “transcript pattern” in the cross-platform comparison evaluation to remove potential confounding factors. The collection of a common set of transcripts at probe/exon level was referred to as a transcript pattern. When cross-platform differences are observed without this level of stringent comparison, it is unknown whether said differences are at the platform level, or simply because different transcripts are being measured. Use of the transcript pattern enables more of an “apples-to-apples” comparison and reduces potential transcript signal bias as a confounding factor to the analysis and interpretation.

With this multi-faceted and bioinformatically rigorous approach, the data presented here both confirmed and expanded upon prior studies. A number of these studies illustrated superior performance of RNA-seq over microarrays in dynamic range and number of differential genes with a comparator of Affymetrix microarrays [5, 7–9]. Our data confirmed the prior findings and demonstrated similar findings for the Illumina array platform as well. However, there was a notable exception to this trend. The Agilent platform modified with Kreatech labeling performed quite comparably to both RNA-seq protocols, as tested, in most categories and was marginally superior in some cases, especially in the overall assessment of fold-change. Furthermore, the evaluation of fold-change accuracy showed that 4 platforms (Agilent, Gene1.0, and 2 RNA-seq methods) had over 60-80 % concordance, as measured, with the gold standard TaqMan assays (Fig. 5k). In general, our results demonstrated a fair degree of overall correlation between all platforms and yet revealed that, to varying degrees, three platforms, both RNA-seq protocols and the Agilent microarray, outperformed the remaining three microarray platforms in most categories of comparisons.

While we observed the overall differences in platform performance, there were several platform-to-platform issues that should be discussed further. First, we observed a very low signal fidelity to sample titration in data generated by Illumina microarrays. When looking deeper into this issue, we found that this happened just in the subsets of low-mid expression genes. Illumina arrays showed a 10–15 % of the low-mid expressers that had a coefficient of correlation greater than absolute 0.5 in the regression analysis between signal intensity and sample titration but Agilent platform was at 20–36 %. Conversely, the two platforms were quite comparable for high expressers (41.3 vs. 40.6 %). In terms of array design and scanner attributes, Illumina arrays appear to have smaller spots and a smaller dynamic signal range than other microarray platforms. If the error in signal measurement does not compress with the overall signal, it may lead to reduced array sensitivity and the ability to capture subtle differences of transcript abundance in titrated samples [18]. Somewhat expectedly, we also identified a correlation between overall fold-change magnitude and broader observed signal/count ranges, particularly the inter-quartile range (IQR), at the platform level. Second, we noted 20 cases in which genes showed a substantial overestimation of down-regulation in terms of BMO/AGO fold-change by RNA-seq RiboZero as compared to TaqMan. Third, the data illustrated that ClonTech provided a 64 % increase in measured fold-change magnitude compared to RiboZero (8.88 vs. 5.42 folds) in the refined 7034 subset data (Fig. 3d). Though it has not been confirmed, we suspect that both of the above issues may be due to a higher degree of read variation for low-abundance transcripts as measured by RiboZero. While, in theory, RiboZero reagents should remove all ribosomal RNAs, in practice it is not always as efficient as is desired [19]. Furthermore, there are more RNA species captured, in abundance, by ribosomal depletion methods and the combination of these events may lead to a dilution of the sequenced reads across a greater total number of RNAs resulting in the hypothesized increase of low-abundance variation. Though it should be noted that this reasoning is speculation on our part and has not been confirmed.

Lastly, we have noted that Affymetrix whole-transcript microarray platforms (Gene1.0 and HTA2.0) yielded the lowest magnitude of fold-change estimates while 3′ biased Agilent chips produced the highest fold-change magnitude, although the same 3′ biased Illumina chips failed to generate comparably high fold-change estimates. This may lead to a confusing concept that 3′ biased arrays generally have greater signal dynamic range than whole-transcript chips. Actually it is not a 3′ versus whole-transcript design issue, but a far more complex issue that involves varying RNA amplification and/or probe hybridization efficiencies, different sets of alternatively spliced transcripts in measurement, and different infrastructures of microarrays (spot size, spot distance, background composition, etc.) [20, 21]. As a result of this high degree of complexity, we sought to introduce the concept of “transcript pattern” to minimize other confounding factors in a multi-platform comparison such as this and any other applicable validation experiments involving 2 or more platforms. With the use of transcript pattern approach, therefore, the observed difference in fold-change magnitude across platforms should largely be attributed to the differences in array inherent characteristics.

## Conclusions

Prior studies have made fundamental comparisons between RNA-seq and microarrays with focus on dynamic range of raw signal/read values and overlap in observed differentially expressed genes. The present study has expanded the foundation laid in the prior studies by looking at the correlations of multiple platforms with regard in signal-to-background ratios, signal/read fidelity to sample titration, and platform performance for providing accurate fold-change estimates. Our results confirmed many of the general findings of the prior studies. RNA-seq methods tend to outperform microarray platforms in regards to certain features such as dynamic range (signal). However, we did observe that the custom protocol Agilent platform performed quite comparably to RNA-Seq, at the given read depth, in virtually all categories. This is presumably due to the increased dynamic range of the platform, suggesting that small improvements in this key feature may put microarrays on a more even footing with RNA-Seq overall. Furthermore, even in the presence of compressed signal range and fold change estimates, the different Affymetrix platforms performed well in the categories of titration and TaqMan fidelity, as compared to Agilent and the RNA-Seq protocols. In addition, the concept of a “transcript pattern” was employed to better enable an “apples-to-apples” comparison of all platforms. As may have been anticipated, the most significant differences observed in the use of common transcript pattern probes, when compared to the larger RefSeq defined transcript set, was noted in the assessment of fold changes and correlation. This was particularly true for the Agilent microarrays and CloneTech RNA-Seq. These data are suggestive that utilization of transcript patterns may be useful for minimizing potential confounding factors in multi-platform comparisons of this nature.

## Methods

### RNA samples

Two original total RNA samples were utilized in this study: one was a pool of RNA isolations from 4 human bone marrow specimens (labeled as BMO) and the other was a Universal Human Reference RNA from Agilent (labeled as AGO). The human bone marrow samples were collected from normal donors with written consent form for use in this study, and these individual RNA samples showed very similar expression profiles in a prior unrelated project. The AGO was made from multiple human cell lines. Both samples had a RNA integrity number (RIN) over 9 assessed by Agilent Bioanalyzer 2100 (Santa Clara, CA). In order to evaluate the ability of a specific platform to detect subtle changes in gene signals and differentials, we generated 3 new samples from the original AGO and BMO RNA samples by mixing the original pure BMO into AGO at a ratio of 1:4, 1:16, and 1:64, and the 3 new RNA samples were labeled as AG1BM4, AG1BM16, and AG1BM64, respectively. These 5 samples were processed in 1–3 replicates (duplicate on average) for microarrays and RNA-seq analyses (Table 2). This study has been approved by the Institutional Review Board of the Human Research Protection Office at the Washington University.

### Microarray platforms and assays

Four microarray platforms were evaluated, including (1) Agilent Whole Human Genome Microarray 4x44K v1 (symbolized as AGLN hereinafter), (2) Illumina HumanHT-12 v4 Expression BeadChip (ILMN), (3) Affymetrix Human Gene 1.0 ST Array (Gene1.0), and (4) Affymetrix Human Transcript Array 2.0 (HTA2.0). We followed vendors’ recommended standard protocols to process the samples for HTA2.0 and ILMN arrays but substituted RNA amplification system with NuGEN kit for Gene1.0 and cDNA labeling system with Kreatech kit for AGLN arrays (see following). For AGLN slides, 200 ng of starting RNAs were amplified with MessageAmp™ II aRNA Amplification Kit (Ambion/Life Technologies, Grand Island, NY), 3,000 ng of amplified aRNA were labeled with Kreatech ULS™ Fluorescent Labeling Kit (with Cy5) (Kreatech Biotechnology B.V., Amsterdam, Netherlands), and 2,055 ng of cy5-labeled aRNA were hybridized. For ILMN BeadChips, 100 ng of starting RNAs were amplified with the MessageAmp™ aRNA TotalPrep kit (Ambion/Life Technologies) and 750 ng of biotin-labeled cRNA were hybridized. For Gene1.0 chips, 50 ng of starting RNAs were amplified with NuGEN Ovation PicoSL WTA V2 kit (NuGEN Technologies Inc., San Carlos, CA) and 2,500 ng of biotin-labeled cDNAs were hybridized. For HTA2.0 chips, 100 ng of starting RNAs were amplified with Ambion WT-plus amplification kit (Ambion/Life Technologies) and 5,000 ng of biotin-labeled cDNAs were hybridized.

### Microarray data processing

We process the raw data with vendors’ default settings. For AGLN, the Agilent Feature Extraction tool (v11.5.4) was used to generate raw data at probe level, rProcessedSignal was used in normalization and data analysis processes, and rIsWellAboveBG parameter was used to mark probes as “detected” or “un-detected”. For ILMN, Illumina GenomeStudio software (v2011) was used to export background-subtracted raw data and detection p-values at both probe and gene levels, and a *p* < 0.05 was set to make “detected” calls. The AGLN and ILMN probe- and gene-level raw data were normalized by most commonly utilized “quantile” method. For Gene1.0 and HTA2.0, Affymetrix Expression Console (v1.3) was used to generate default raw data at both probeset and transcript cluster levels, and the raw data were RMA background corrected, median polish summarized and quantile normalized. Detection p-values were calculated for all probesets on Gene1.0 and HTA2.0 chips, a *p* < 0.01 was set to make “detected” calls at the probeset level.

### Mapping and re-annotation of microarray probes

In order to enable an exact comparison of transcript abundance across platforms, we first re-annotated vendors’ probes (probesets for Affymetrix chips) to correct any outdated gene information and to minimize probe(set)s with non-specific hybridization. Ensembl release version 72 (R72) of human reference transcriptome and genome databases were used to make new probe(set) annotations. For AGLN, ILMN and Gene1.0, probe(set) sequences provided by vendors were mapped onto Ensembl R72 genome using novoalign tool (Novocraft Technologies, Selangor, Malaysia) to define probe(set) coordinates. For HTA2.0, probeset coordinates provided by the vendor were used to retrieve probeset sequences from Human Genome hg19 database. We then used the retrieved probeset sequences to map the probesets onto Ensembl R72 reference genome. Any probe(set)s with multiple alignments or partial alignments to the genome were omitted in downstream analysis. Finally, the uniquely aligned probe(set)s derived from all platforms were intersected with Ensembl R72 transcriptome using BEDtools [22] to obtain Ensembl gene ID, transcript ID, exon ID, and corresponding RefSeq gene names for each probe(set). With this information, we were able to identify a subset of probe(set)s of specific genes that targeted a common set of transcripts across all 4 microarray platforms, which we refer as to “transcript pattern” (Fig. 1). In the RNA-seq, it was a subset of exons of specific genes that shared a common set of transcripts and were targeted by microarray probe(set)s.

### RNA-seq assays

Two different library preparation protocols were tested: oligo-d(T)-priming based Clontech SMARTer Ultra Low RNA Kit (hereafter labeled as “ClonTech”) for Illumina® Sequencing from Clontech Laboratories (a Takara Bio Company, Mountain View, CA), and random priming based Ribo-Zero™ rRNA Removal Kits (labeled as “RiboZero”) from EpiCentre (an Illumina company, Madison, WI). A starting amount of 10 ng RNAs was processed in ClonTech assays, and 1 ug were input with RiboZero protocols. For ClonTech libraries, amplified cDNA was sheared using a Covaris E210 instrument. RiboZero depleted RNA was chemically fragmented, then made into cDNA with Superscript III (Life Technologies) and random hexamers followed by a second strand reaction. cDNA from both methods was then end repaired, A tailed, and standard Illumina adapters were ligated on. Libraries were then amplified with primers to incorporate a unique index to each sample. In pooling of multiple libraries for running on a single lane, equal amount of library mass was determined by Qubit reading and Bioanalyzer for the 5 RNA libraries. Lastly, pooled libraries were amplified with Illumina® TruSeq™ Cluster kits and sequenced with Illumina® sequencing primers on Illumina® HiSeq2500™ next-generation sequencing system as a high output single read 50 cycle run (Illumina Inc., San Diego, CA).

### Processing of RNA-seq reads

An average of 36.7 (range 18.1–53.0) and 27.9 (range 22.9–43.9) million 1x50 bps sequence reads were obtained from the 5 RNA samples with ClonTech and RiboZero protocols, respectively. Read sequences in fastq files were aligned to Ensembl R72 whole-genome with TopHat version 2.0.8 using Bowtie2 version 2.1.0 for quantification. Both exon- and gene-level count data were generated using scripts “dexseq_count” and “htseq-count”, respectively, in a Python package “HTSeq” [23] for differential expression analysis. To avoid a denominator being 0 in calculation of ratio values, we added 1 count to every data point. Using an R package “edgeR” from Bioconductor [24, 25], the raw count data at exon- and gene-levels were normalized by library size, and values for the 4 ratios (AG1BM4 vs. AGO, AG1BM16 vs. AGO, AG1BM64 vs. AGO, and BMO vs. AGO) were calculated with normalized counts per million (cpm) data. In platform comparisons, we retained those genes with at least 1 count in any of the 5 samples, and defined “detected” genes at a normalized counts per million (cpm) > 0.25 in 1 of the 5 samples.

### Evaluation of platform capability for providing differential estimates

One primary goal of our study is to evaluate the platform performance in magnitude of differential expression, i.e. fold-change. Therefore, ratios of AG1BM4 vs. AGO, AG1BM16 vs. AGO, AG1BM64 vs. AGO, and BMO vs. AGO were first calculated utilizing signal and read count data. Furthermore, these were completed at the level of the entire data set as well as the transcript pattern derived subset data for each platform. Ratios were transformed to absolute fold-changes, i.e. ratios for up-regulated genes remained the ratio per se, and any ratios less than 1 for down-regulated genes were converted to its reciprocal values (i.e. switching the nominator and denominator to make the ratio greater than 1). Next, the absolute fold-change values in a data set were averaged to reflect the platform capability for providing differential expression estimates.

### TaqMan qRT-PCR

TaqMan assays were run for 48 genes on each of the 5 RNA samples that were used in microarray and RNA-seq assays. These 48 genes were profiled as part of an unrelated project. The assays were carried out using microfluidic-based digital PCR system with 48.48 Dynamic Arrays (Fluidigm Corp. San Francisco, CA). In brief, 500 ng total RNAs were subjected to specific target amplification (STA) using High Capacity cDNA Reverse Transcription Kit and TaqMan PreAmp Mastermix (Applied Biosystems/Life Technologies, Foster City, CA), and then each cDNA sample was diluted 1part sample into 4 parts low ethylenediaminetetraacetic acid (EDTA) DNA suspension buffer. The qPCR reaction was prepared with diluted cDNA samples, 20X Gene Expression Sample Loading Reagent (Fluidigm Corp.), TaqMan Universal PCR Master Mix (Applied Biosystems), and Dynamic Array Assay Loading Reagent that contained 9 μM of each primer and 2 μM of the probe (Fluidigm Corp). The qPCR program was as following: 50 °C for 2 min, 95 °C for 2 min, 40 cycles of 95 °C for 15 s and 60 °C for 1 min. Post-PCR analysis was performed to mark failed samples with a Ct value greater than 40 in over 50 % of replicates and to remove unreliable samples with coefficient of variance greater than 30 % in Ct values across replicates. Relative gene expression level was calculated by delta-delta Ct method [26].

### Concordance in fold-change between TaqMan and microarray/RNA-seq protocols

In determination of fold-change concordance between TaqMan (X) and microarrays/RNA-seq protocols (Y), 4 qualitative evaluations were assigned to each comparison: compressed, opposite, overestimate, and concordant. When two compared fold-changes are in the same direction but the ratio of X/Y greater than or equal to 2, a value of “compressed” is assigned. Similarly, if the fold-change ratio of X/Y is less than or equal to 0.5 the comparison is deemed “overestimate”. Fold-change ratios between these values are deemed “concordant”. When two fold-changes are not in the same direction and either of them is greater than 2 or less than 0.5, the comparison is determined to be “opposite”. Concordance rates were calculated by number of genes with “concordant” and “overestimate” calls divided by the total genes analyzed.

### Data access

All microarray and RNA-seq data are accessible in the GEO database, with accession number GSE66590 for RNA-seq_RiboZero, GSE66592 for RNA-seq_ClonTech, GSE66614 for Illumina_HT12, GSE66626 for Agilent_4x44K, GSE66628 for Affymetrix_Gene1.0 and GSE66648 for Affymetrix_HTA2.0. A SuperSeries accession number GSE66649 is created for access to all these data in the whole project.

## Additional files

Additional file 1: Figure S1. Box-Whisker plot for illustration of fold-change variability in each platform. (A) and (B) are for fold-change data in the 4 sample titrations in entire set and transcript pattern (TP) defined 7,034 RefSeq genes subset data, respectively; (C) and (D) are for fold-change data at overall platform level in the entire set and TP-defiend subset data, respectively. The frame boxes are the inter-quartile range (i.e. 25 % to 75 %). (TIFF 867 kb) Additional file 2: Figure S2. Overall average absolute fold-change comparisons across platforms with entire set and transcript pattern (TP) defined subset data, with a focus on the effect of Affymetrix “Signal Space Transformation” (SST) algorithm on the overall platform fold-change magnitude. The SST, in conjunction with the regular robust multiple-array average normalization method (SST-RMA, the dark blue bars), was able to improve the fold-change in the HTA2.0 arrays, and provided a 2-5x greater fold-change estimates overall, as compared to conventional data processing method (regular RMA, the light blue bars). (TIFF 491 kb) Additional file 3: Figure S3. Scatter plot of log2Ratio data between all platforms (except for those compared to AGLN in Fig. 4): (A) 15,442 common RefSeq genes and (B) transcript pattern restricted 7,034 RefSeq genes, each against AGLN. The dotted green lines are trend lines by linear regression, and the red lines are diagonal lines of the frames. The deviation of green lines from red lines indicates the degree of fold-change compression that can be quantified by slope values in the equations. (TIFF 1212 kb)

## Abbreviations

AGO: Agilent Universal Human Reference RNA
BMO: normal donor bone marrow RNA
AGLN: Agilent Human 4x44K microarray platform
ILMN: Illumina Human HT12 microarray platform
Gene1.0: Affymetrix Human Gene Array ST 1.0
HTA2.0: Affymetrix Human Transcriptome Array 2.0
ClonTech: A RNA-seq protocol utilizing oligo-d(T)-priming based Clontech SMARTer Ultra Low RNA Kit
RiboZero: Another RNA-seq protocol utilizing random priming based Ribo-Zero™ rRNA Removal Kit
qRT-PCR: quantitative reverse transcription polymerase chain reaction
cpm: counts per million (i.e. RNA-seq reads normalized by library size)
