# Supplementary figures and images for: Multi-platform assessment of transcriptional profiling technologies utilizing a precise probe mapping methodology
Source: BMC Genomics. 2015 Sep 18;16(1):710. doi: 10.1186/s12864-015-1913-6 (PMC4575490; doi:10.1186/s12864-015-1913-6)

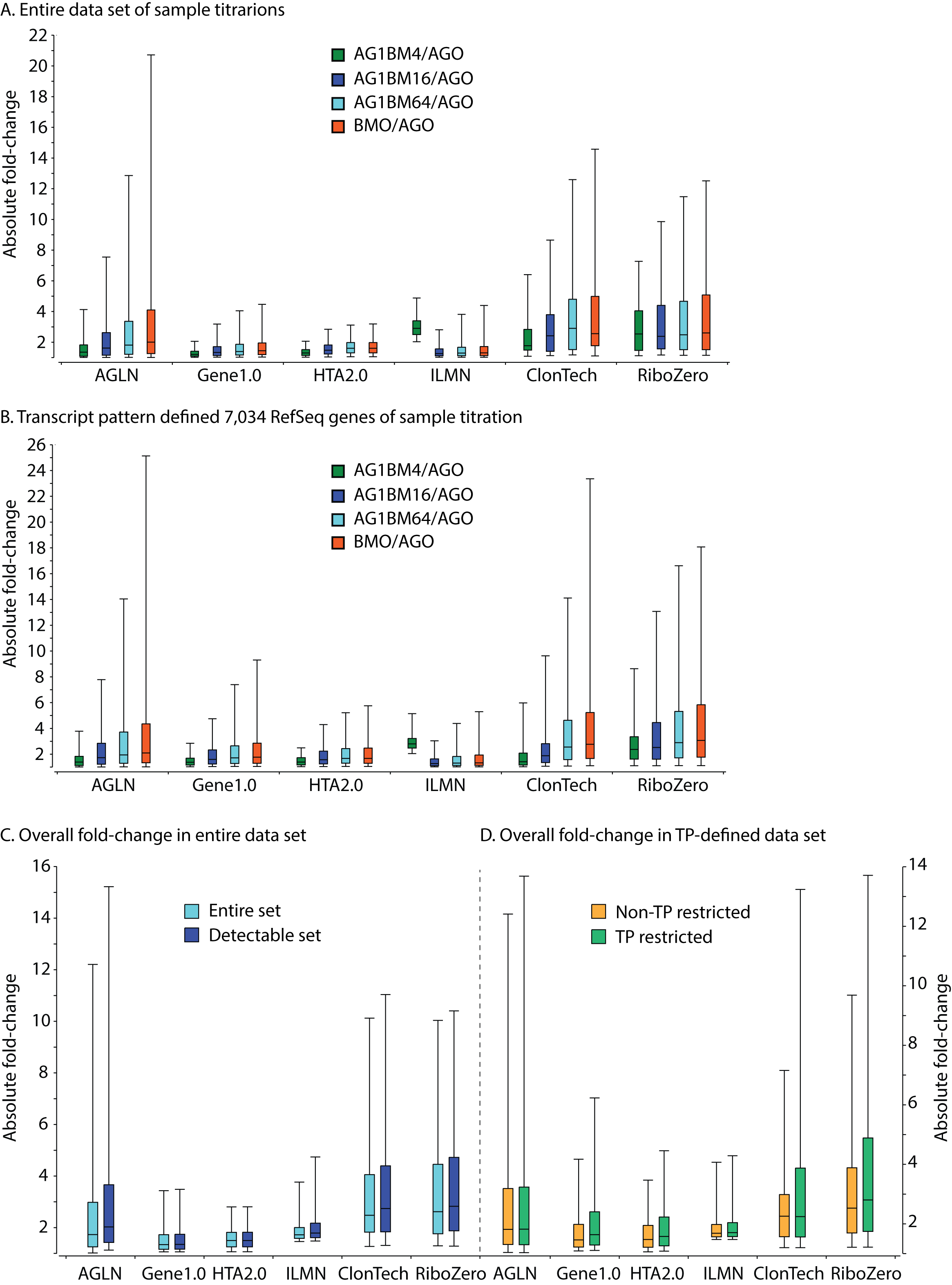

Supplement: Additional file 1: Figure S1. — Box-Whisker plot for illustration of fold-change variability in each platform. (A) and (B) are for fold-change data in the 4 sample titrations in entire set and transcript pattern (TP) defined 7,034 RefSeq genes subset data, respectively; (C) and (D) are for fold-change data at overall platform level in the entire set and TP-defiend subset data, respectively. The frame boxes are the inter-quartile range (i.e. 25 % to 75 %). (TIFF 867 kb) [file 12864_2015_1913_MOESM1_ESM.tif]

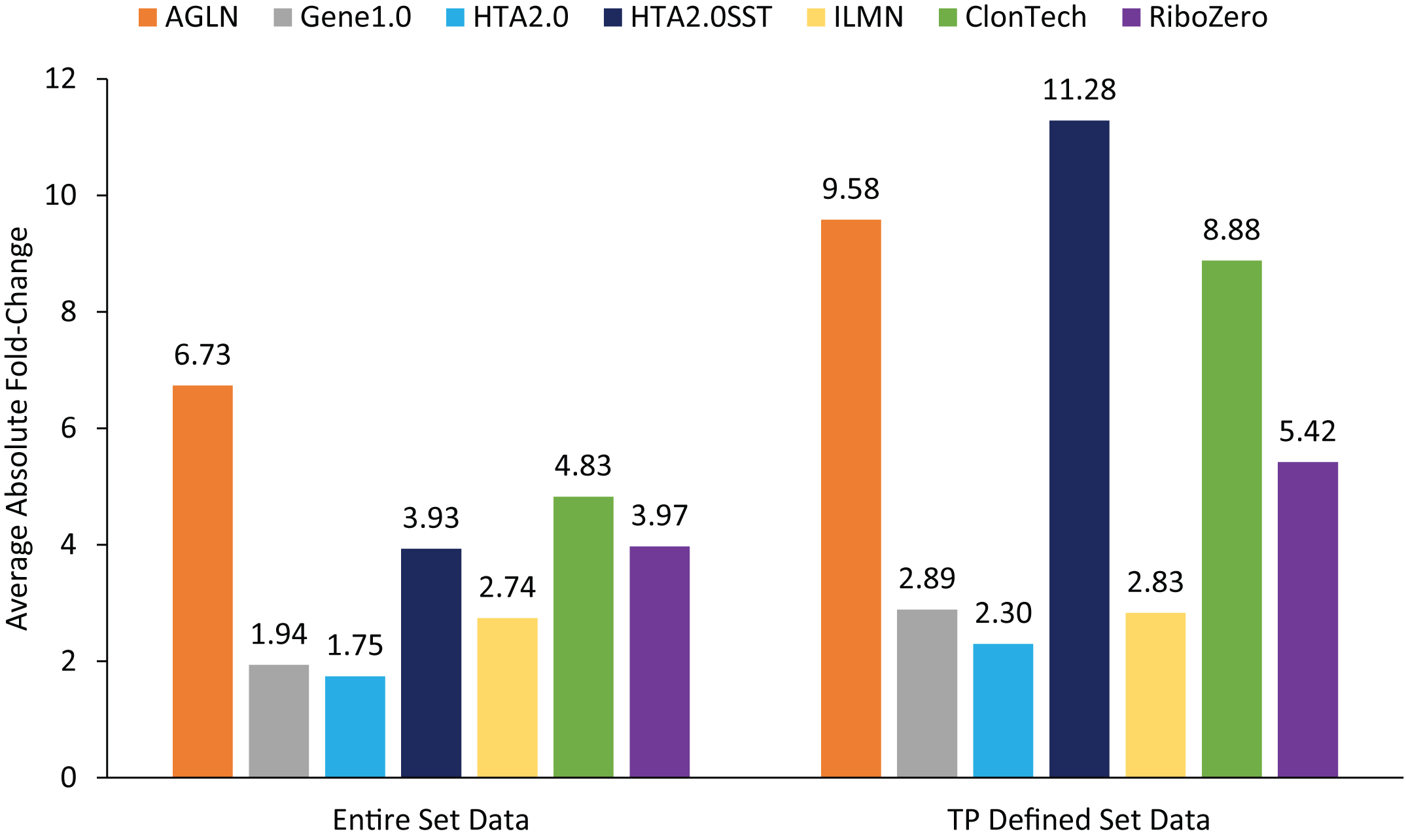

Supplement: Additional file 2: Figure S2. — Overall average absolute fold-change comparisons across platforms with entire set and transcript pattern (TP) defined subset data, with a focus on the effect of Affymetrix “Signal Space Transformation” (SST) algorithm on the overall platform fold-change magnitude. The SST, in conjunction with the regular robust multiple-array average normalization method (SST-RMA, the dark blue bars), was able to improve the fold-change in the HTA2.0 arrays, and provided a 2-5x greater fold-change estimates overall, as compared to conventional data processing method (regular RMA, the light blue bars). (TIFF 491 kb) [file 12864_2015_1913_MOESM2_ESM.tif]

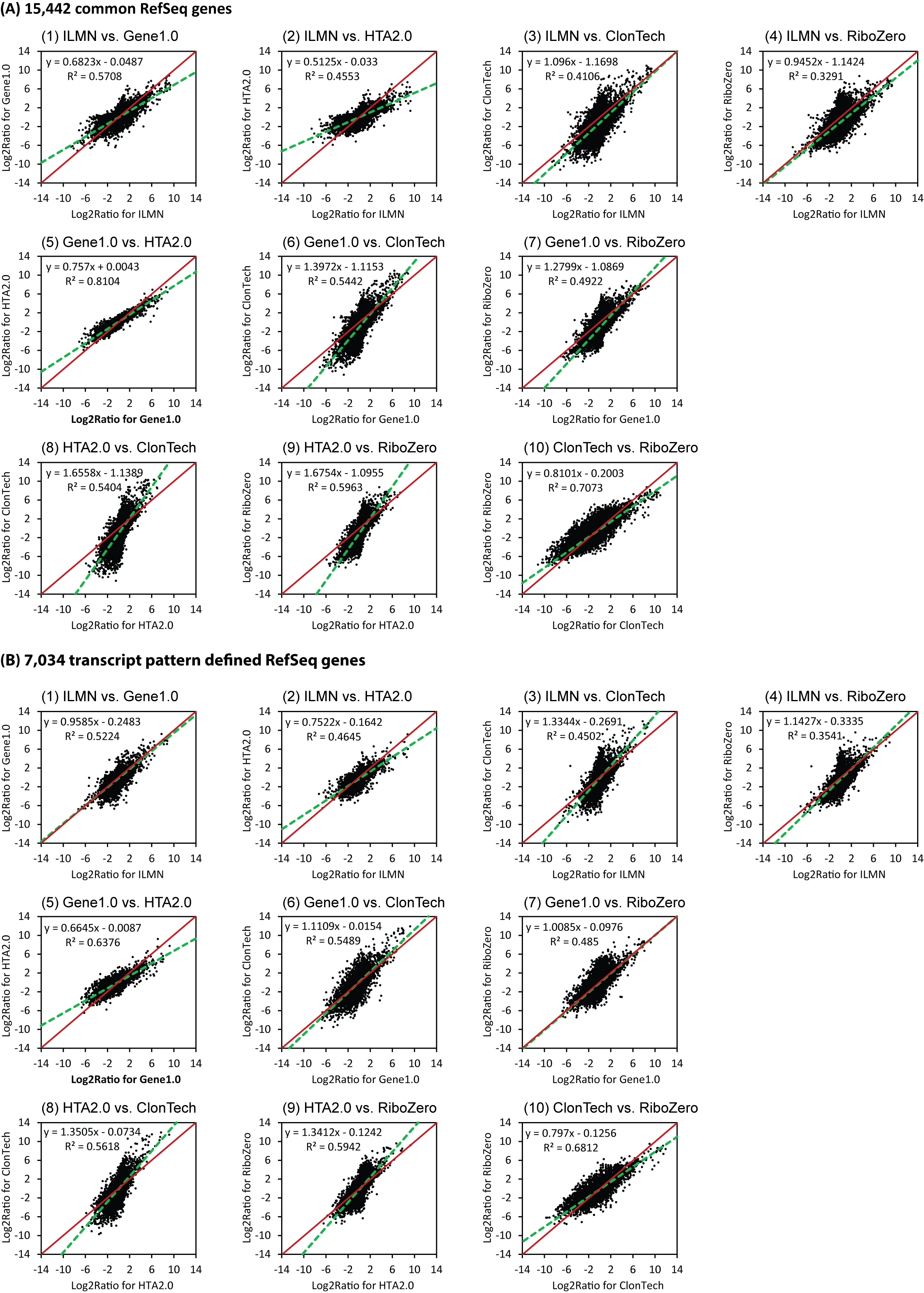

Supplement: Additional file 3: Figure S3. — Scatter plot of log2Ratio data between all platforms (except for those compared to AGLN in Fig. 4): (A) 15,442 common RefSeq genes and (B) transcript pattern restricted 7,034 RefSeq genes, each against AGLN. The dotted green lines are trend lines by linear regression, and the red lines are diagonal lines of the frames. The deviation of green lines from red lines indicates the degree of fold-change compression that can be quantified by slope values in the equations. (TIFF 1212 kb) [file 12864_2015_1913_MOESM3_ESM.tif]
